# Incremental dialysis: two complementary views

**DOI:** 10.1093/ckj/sfae020

**Published:** 2024-02-05

**Authors:** Debaters: Francesco Gaetano Casino, Mariana Murea, Moderators: Jürgen Floege, Carmine Zoccali

**Affiliations:** Department of Internal Medicine, Division of Nephrology, Hospital Madonna delle Grazie, Matera, Italy; Department of Internal Medicine, Section on Nephrology, Wake Forest University School of Medicine, Winston-Salem, NC, USA; Division of Nephrology and Rheumatology, RWTH Aachen University Hospital, Aachen, Germany; Renal Research Institute NY, USA; BIOGEM, Ariano Irpino, Italy; IPNET, Reggio Cal, Italy

**Keywords:** equivalent renal urea clearance (EKRU), haemodialysis, incremental haemodialysis, residual kidney urea clearance (Kru), standard Kt/V (stdKt/V)

## Abstract

Franco Casino and Mariana Murea discuss today's knowledge about the ‘incremental dialysis’ concept. Franco Casino frames the problem by saying that, in the presence of substantial residual kidney function, kidney replacement therapy can begin with low doses and/or frequencies, to be gradually increased to compensate for any subsequent losses of residual kidney function, keeping the total clearance above the minimum levels of adequacy. He remarks that studies so far have documented that this approach is safe. He recognizes that adequate randomized controlled trials (RCTs) are necessary to confirm the safety and simplify and standardize the practical aspects of this approach. Mariana Murea objects that most of the evidence gathered so far primarily derives from retrospective and observational studies, which can be influenced by socioeconomic constraints. She argues for the need for RCTs to provide compelling empirical evidence on the efficacy of incremental dialysis. Nephrologists are still reluctant to adopt this approach for various reasons, including unfamiliarity with the method, lack of practical guidance and financial disincentives. Several countries have ongoing or planned RCTs comparing incremental dialysis with conventional dialysis. These trials can shift the haemodialysis paradigm if they validate the safety and effectiveness of this approach. The moderators believe that the results of ongoing trials must be carefully interpreted, and further validation may be needed across different patient populations or healthcare settings. The ultimate goal is to gather robust evidence that could lead to widespread adoption of incremental haemodialysis, optimizing treatment, reducing overtreatment, preserving resources and improving patients’ quality of life.

More than 60 years after the introduction of kidney replacement therapy (KRT), there is still uncertainty about the most appropriate approach to start it. The ‘conventional’ approach of starting dialysis at full dose was adopted essentially for practical reasons, without being based on any randomized controlled trial (RCT) and, on the other hand, evidence on the safety and efficacy of the incremental approach is still sparse [1]. Franco Casino and Mariana Murea provide complementary views of the problem in this review. Franco Casino highlights the concept of incremental dialysis has taken ground in the realm of peritoneal dialysis but has resistance to being accepted in patients treated with haemodialysis (HD), while Mariana Murea stresses that a randomized controlled trial (RCT) remains a clinical research and public health need.

## THE PRO-VIEW

In the view of Franco Casino, incremental initiation appears to be the most logical way of initiating KRT, because it is obvious that less replacement is needed at the beginning, when residual kidney function is usually present, compared with later stages when residual kidney function may be very reduced or absent. Although adequate RCTs are needed before the incremental approach can become the standard way to initiate KRT, it cannot be ignored that three recent systematic reviews [2–4] found results almost uniformly favourable to incremental HD. The first systematic review [2] analysed all available observational studies evaluating at least one of the following outcomes: all-cause mortality, residual kidney function loss and time to full-dose dialysis. There were 22 studies, 15 in HD and 7 in peritoneal dialysis (PD). Incremental PD was defined as <3 daily dwells in continuous ambulatory PD and <5 sessions per week in automated PD, while incremental HD was defined as <3 HD sessions/week. When compared with the full dialysis dose, incremental dialysis (incremental HD or incremental PD) had an overall mortality risk of 1.14 [95% confidence interval (CI) 0.85–1.52] and were associated with a lower mean residual kidney function loss (difference −0.58 mL/min/month, 95% CI 0.16–1.01). Overall, the time to initiation of full-dose dialysis was 12.1 months (95% CI 9.8–14.3), with no difference between incremental HD and incremental PD. This systematic review concluded that ‘Incremental dialysis allows longer preservation of residual kidney function, thus deferring full-dose dialysis by about 1 year in HD and PD with no increase in mortality risk’ [2].

In contrast to the first review [2], which analysed observational studies involving incremental PD and/or incremental HD, the second review [3] focused only on incremental HD; in addition to observational studies, it included two recently published pilot feasibility RCTs [5, 6]. Incremental HD was defined by <3 sessions per week or lasting <3.5 h with standard thrice weekly treatment. The primary outcome was mortality; secondary outcomes included treatment-emergent adverse events, loss of residual kidney function, quality of life and cost-effectiveness. Twenty-six studies, comprised of 24 cohort studies and 2 pilot feasibility RCTs that totalled 101 476 participants, were analysed. No differences in mortality were found between conventional-start and incremental-start HD (hazard ratio = 0.99; 95% CI 0.80–1.24). Cohort studies suggested similar hospitalization rates, while the two pilot feasibility RCTs suggested reduced hospitalization risk with incremental HD initiation. Data on other treatment-emergent adverse events and quality of life were limited. However, one of the two pilot feasibility RCTs [6] found that neither fluid overload nor hyperkalaemia episodes differed significantly between the two arms; on the contrary, bicarbonate levels were significantly lower in incremental HD patients, indicating that supplementation may be required. An unexpected result of this pilot RCT [6] was the absence of signals in favour of better preservation of residual kidney function by incremental HD compared with the conventional regimen, in contrast to the findings of observational studies. However, as the authors acknowledge, ‘this may reflect a lack of power’ [6], and therefore further studies are required. The conclusions were: ‘Confirmation of the safety of incremental HD initiation in a large randomized controlled study would support its widespread adoption, particularly given the apparent cost benefit’ [3].

The third review included a total of 36 original articles (138 939 participants) [4]. The mortality rate and cardiovascular events were similar between incremental and conventional HD with odds ratios (OR) of 0.87 and 0.67, respectively. However, hospitalization rates and loss of residual kidney function were significantly lower in patients treated with incremental HD (OR 0.54 and 0.31, respectively). Vascular access complications, hyperkalaemia and volume overload were not statistically different between groups.

In closing, Franco Casino points out that appropriate RCTs are still needed to simplify the practical management of incremental dialysis, which is currently relatively challenging, especially in HD. The National Kidney Foundation Kidney Disease Outcomes Quality Initiative clinical practice guidelines have already incorporated the concept that the dose and/or frequency of PD or HD may be lower at the initiation of dialysis, in the presence of substantial residual kidney function, expressed by residual kidney clearance of urea (Kru), and that the dialysis dose should be progressively increased to compensate any subsequent reduction in Kru [7, 8]. The basic rule is that the weekly sum of Kru and dialytic urea clearance (Kd) should always be at least equal to a pre-established adequate level and that any reduction in Kru should be replaced by the same weekly amount of Kd [7, 8]. In the case of PD, its adequacy must be expressed by a weekly Kt/V ≥1.7, and the weekly PD dose (Kt/V) to be administered is obtained as follows: Kt/V = 1.7 – Kru × 10 080/V, where V is the patient's urea distribution volume (V), usually estimated with the Watson formula [7].

**Figure 1:**
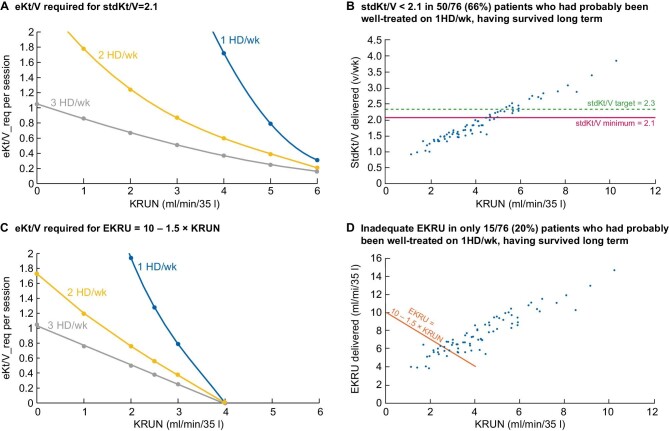
Comparison between stdKt/V and EKRU. (**A, C**) The curves predicting the required dialysis dose (eKt/V_req) to obtain stdKt/V = 2.1 volumes/week or EKRU = 10–1.5KRU mL/min/35 L, on 1, 2 and 3 sessions per week regimens, respectively. (**B, D**) The distribution of delivered stdKt/V and EKRU, respectively, in a group of 76 patients on once-weekly HD: 66% of them should be classified as inadequately treated according to the stdKt/V criterion but only 20% of them according to the EKRU criterion. Symbols: 1HD/wk = 1 HD session per week; 2HD/wk = 2 HD sessions per week; 3 HD/wk = 3 sessions per week; eKt/V = equilibrated Kt/V; KRUN = normalized Kru (mL/min/35 L).

**Figure 2:**
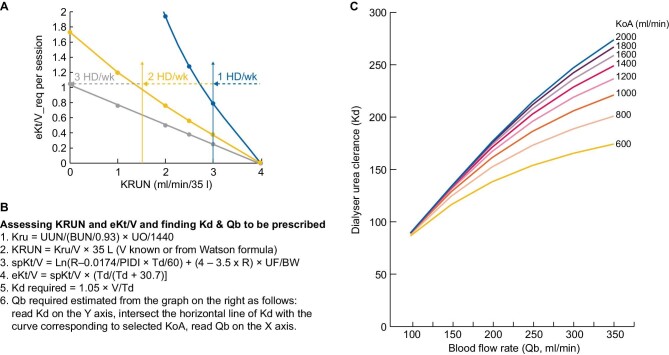
Simplifying the prescription and evaluation of incremental HD. The graph in (**A**) is the same as in Fig. 1C, with the addition of a dotted horizontal line which corresponds to a constant eKt/V of 1.05, which crosses the once-weekly treatment line at KRUN around 2.8 mL/min/35 L, and therefore, for greater safety, a KRUN limit of 3 mL/min/35 L can be accepted. Similarly, a KRUN limit of 1.5–2 mL/min/35 L can be accepted for twice-weekly treatment. The equations in (**B**) are taken from reference [9], with slight modifications. The graph in (**C**) was also obtained with the equations reported in reference [9]. Symbols: Kru = kidney urea clearance (mL/min); UUN = urinary urea nitrogen (mg/dL); BUN = blood urea nitrogen (mg/dL); UO = urine output (mL/day); KRUN = normalized Kru (mL/min/35 L); spKt/V = single pool Kt/V; Ln = natural logarithm; R = post-dialysis BUN/pre-dialysis BUN; PIDI = preceding interdialytic interval (days); Td = session length (min); UF = ultrafiltration per session (kg); BW = post-dialysis body weight (kg); eKt/V = equilibrated Kt/V; V = urea distribution volume (mL); Kd = dialyser urea clearance (mL/min); Qb = blood flow rate (mL/min); KoA = dialyser urea mass transfer-area coefficient (mL/min).

In the case of incremental HD, an evidence-based adequacy criterion has not yet been established. Therefore, it can be provisionally based on one of the two available equivalent continuous clearances, i.e. standard Kt/V (stdKt/V) [8] and the new version of equivalent renal urea clearance (EKRU) [9]. The suggested adequate value of stdKt/V is 2.1 units/week [8], and the dialysis dose to be administered is as follows: dialysis stdKt/V = 2.1–Kru × 10 080/V. The formal calculation of stdKt/V requires using the double-pool urea kinetic model; however, a simple formula is also available [8]. Unfortunately, a simple method that provides the precise, adequate dialysis dose (Kt/V) to be prescribed is lacking, and for this reason, many physicians often prescribe a very high dialysis dose—which is not necessarily useful and could actually be harmful for residual kidney function—to guarantee a stdKt/V value not less than 2.1 units/week. A simple tool to meet this need has been published recently [10], a further simplified version of which is being proposed here (Figs 1 and 2). In brief, the suggested minimum adequate value of EKRU, according to the recently introduced ‘variable target model’ [9], which emphasizes the clinical value of Kru, is computed as follows: EKRU = 10–1.5 × KRUN, where KRUN = Kru/V × 35 L [1]. Of note, the above expression implies that each mL/min/35 L of KRUN is worth 2.5 mL/min/35 L of Kd.

The comparison of the graphs in Fig. 1A and C shows that EKRU allows incremental HD for Kru and Kt/V values lower than those required by stdKt/V. Of note, the analysis of Fig. 1B and D supports the hypothesis that stdKt/V overestimates the dialysis requirement in incremental HD, especially in once-weekly HD. In fact, in the incremental HD program adopted by Casino *et al*., stdKt/V would have classified the once-weekly treatment inadequate in 66% of cases in a cohort of 76 patients on once-weekly HD, who had probably been adequately treated, having had, on average, a long survival [11]. Figure 2A illustrates a recent proposal [1] aimed at simplifying the management of incremental HD. Instead of continually adjusting the dialysis dose as KRUN decreases, a constant eKt/V of 1.05 is suggested along with an increase in dialysis frequency as KRUN approaches certain critical levels: once-weekly HD could be maintained until KRUN is ≥3 mL/min/35 L and twice-weekly HD until KRUN is ≥1.5 mL/min/35 L, before switching to the conventional thrice-weekly rhythm. Figure 2B and C simplify, for routine use, the more accurate method introduced by the above tool [10] to calculate KRUN and eKt/V, to evaluate the adequacy of dialysis, to provide the Kd value to be prescribed to obtain eKt/V = 1.05 (Fig. 2B), and the blood flow rate required to achieve the prescribed Kd value (Fig. 2C).

Incremental dialysis is a rational way to start KRT. This is well accepted in PD, but not yet in HD. While awaiting further evidence from ongoing and future RCTs, based on the available data, we can suggest that, in most patients, incremental HD could be started with a once-weekly treatment, then moved to two and then three dialysis treatments per week. Frequent residual kidney function monitoring is necessary, as well as constant maintenance of the adequacy of the overall therapy and not just the dialytic one. In practice, incremental dialysis should be seen as a therapeutic means integrated with the therapy implemented in the conservative phase, which must be substantially maintained, with appropriate adjustments, even in the subsequent dialysis phase.

## THE CON-VIEW

Mariana Murea begins by saying that, like Newton's first law of motion, the widespread practice of conventional HD, consisting of thrice-weekly HD treatment initiation, is a form of clinical inertia. Indeed, a gradual onset and escalation of dialysis sessions, carefully adjusting to a patient's remaining kidney function and comprehensive health status [12], is suggested by an expanding collection of scientific studies [13]. Yet, conventional HD, a practice borne in societies ostensibly unaffected by depletable resources, resembles an object that has steadfastly maintained its trajectory [3].

In the realm of healthcare, the force that can disrupt clinical inertia is usually science—that is, empirical evidence showing an alternative treatment methodology is either superior in effectiveness or similarly effective but less taxing for both patients and society. Empirical evidence–shaping practice primarily falls into two categories: observational studies, with naturally gathered and database-derived results, and RCTs, which systematically confirm hypotheses often derived from observational studies. In the last three decades, over 20 retrospective database-derived and prospective observational studies have reported that less than thrice-weekly HD, compared with conventionally dosed thrice-weekly HD, conferred similar patient survival, similar or better quality of life, and a slower decline in residual kidney function [3]. Two pilot RCTs involving patients with new-onset chronic HD and residual kidney function demonstrated the feasibility of randomization and implementation of an incremental prescription of initial twice-weekly followed by thrice-weekly HD versus conventional HD [5, 6]. While these pilot studies were not powered to compare clinical outcomes, patient safety outcomes such as hospitalization rate were more favourable in the incremental HD group than the conventional group [5, 6].

It is essential to underscore that less frequent HD schedules often included in retrospective and observational studies were predominantly a by-product of socioeconomic limitations rather than clinically guided decisions toward incremental HD [3]. Not only does miscategorization under ‘incremental HD’ distort the interpretation of the results, but it also undermines potential benefits associated with said treatment. Studies explicitly stating socioeconomic factors as the cause for decreased HD frequency found that patients undergoing less than thrice-weekly HD demonstrated a higher prevalence of hospitalization and mortality (Table 1). Interestingly, in some studies, when scarce dialysis resources and socioeconomic dynamics affected HD frequency, medical factors such as age, smoking habits and coexisting diseases—rather than HD frequency—primarily impacted patient outcomes. Conversely, when less than thrice-weekly HD was delivered as a form of incremental HD, in line with preserved residual kidney function and clinical symptomatology, patients exhibited either comparable or superior outcomes relative to their counterparts treated with conventional HD [3].

**Table 1:** A summary of observational studies and RCTs on HD prescription.

(A) Observational studies of less than thrice-weekly HD in settings of socioeconomic limitations		
Study	Cohort	Results
Aoun *et al*. (2022), Lebanon [S1]	76 patients shifted from thrice-weekly to twice-weekly HD as a means of cost savings	After conversion to twice-weekly HD: higher interdialytic weight gains; no differences in serum potassium, haemoglobin or hospitalization rate
Xinghui Lin *et al*. (2012) and (2018), China [S2, S3]	1041 patients on twice-weekly HD and 1531 patients on thrice-weekly HD	Similar survival between the treatment groups. Age, body mass index, serum albumin and weekly Kt/V were predictors of patient mortality
Mukherjee *et al*. (2017), India [S4]	35 patients on twice-weekly HD and 82 patients on thrice-weekly HD	No significant difference in hospitalization or mortality rates between the two groups. Weight gain, ultrafiltration rates, blood pressures and haemoglobin were more favourable in the thrice-weekly patients
Nieves-Anaya *et al*. (2021), Mexico [S5]	44 patients on twice-weekly HD and 44 patients on thrice-weekly HD	Higher rates of undernutrition, volume overload, hospitalization and death in patients treated with twice-weekly HD
Panaput *et al*. (2014), Thailand [S6]	504 patients on twice-weekly HD and 169 patients on thrice-weekly HD	Similar survival rate and times to hospitalization between the treatment groups. Significant predictors for death were serum albumin, current smoking, age and the Index of Coexistent Disease
Stankuviene *et al*. (2010), Lithuania [S7]	121 patients on once-weekly HD, 874 patients on twice-weekly HD and 1433 patients on thrice-weekly HD	Patients treated with twice-weekly HD had higher mortality rate than those treated with thrice-weekly HD
(B) Observational and randomized controlled trials in HD		
Subject studied	Conclusions from observational studies	Conclusions from RCTs
Dialysis dose	More intensive dialysis dose correlates with improved patient outcomes [S8–S10]	There is no significant difference in mortality rates or cardiovascular outcomes between a higher (equilibrated Kt/V 1.53) or lower (equilibrated Kt/V 1.16) dialysis dose [S11]
Haemoglobin target	There is a linear relationship between higher haematocrit levels and a reduced risk of death [S12]	Higher target haematocrit levels lead to a higher mortality rate compared with those achieved with lower target levels [S13]
Statin use	Statis are associated with reduced mortality in patients on dialysis [S14]	Statin use is associated with higher mortality [S15]
Timing of dialysis initiation	Studies before the year 2000: initiating dialysis at an earlier stage improves patient survival rates. Studies after the year 2000: early dialysis initiation is associated with higher mortality rates [S16]	The rate of all-cause mortality is similar whether dialysis is initiated later or earlier [S17]
Diffusion alone vs diffusion and convection	Results are varied, showing either improved patient survival, or no discernible difference, when comparing haemodiafiltration with HD [S18]	Mixed results, showing either no significant difference or better all-cause or cardiovascular mortality between post-dilution high-volume haemodiafiltration and HD [S19, S20]

Differences in effect estimates between observational studies and randomized controlled trials in nephrology have been studied by Kimachi *et al*. [S21].

Supplementary references [S1] to [S21] are listed in the Supplementary Material.

This scientific knowledge, substantial as it is, has not swayed the prevailing practices of conventional HD [14]. Nevertheless, this might not come as a surprise. To extend the metaphorical analogy, the effectiveness of a force in changing an object's trajectory depends on several factors, including any existing or opposing forces, its point of application, the object's mass and—not least—the magnitude of the force. Let us realize that opposing forces in the field of incremental HD are numerous and powerful [15]. Due to unfamiliarity and limited practical exposure, nephrologists trained under the tenet of conventional HD often find it challenging to adopt alternative models like incremental HD. Physicians might hesitate to provide less HD even when it could be sufficient, anticipating that patients might decline additional HD if recommended it later. While this possibility exists, its prevalence has not been quantified by existing registry data studies. Prospective data collection in RCTs will clarify the extent and clinical implications of patients’ fidelity to transitioning from less to more frequent HD.

In its point of application, unlearning a decades-long doctrine of conventional HD is a complex undertaking. This is further exacerbated by additional time demands for healthcare professionals necessitated by the individualization of HD prescriptions. Although recent guidelines underscore the significance of residual kidney function for patients, practical guidance towards incremental HD implementation remains scant. Existing quality metrics not only do not require reporting of residual kidney function but they enable the maintenance of predetermined dialysis dosing of thrice-weekly HD treatments. Finally, the financial aspects of medical care can determine the success or failure of new treatment models, regardless of the scientific principles involved. A downstream decrease in dialysis revenue from incremental HD will undoubtedly hinder its broad acceptance. In regions where medical practice is driven by fee-for-service, a profound alteration in the reimbursement algorithm is necessary. Thus, analogous to moving a large mass, effecting new behaviour throughout the dialysis workforce needs a significant force to bring about meaningful change.

**Figure 3:**
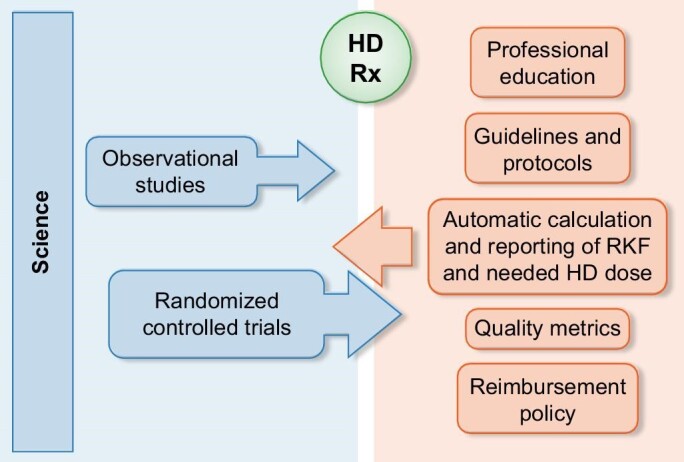
Forces and counterforces that shape the prescription of incremental HD.

What force could catalyse a domino effect, enabling widespread implementation of incremental HD? The answer, proposed by many, points towards the compelling methodology of RCTs [8], dubbed as the ‘gold standard’ in health research. Clinical trials contrasting incremental and conventional HD are underway or imminent in eight countries spanning three continents (Table 2) [16–18]. RCTs can critically evaluate the established normalization of conventional HD by assigning patients with new-onset chronic dialysis and ongoing residual kidney function to either conventional or incremental HD. The potential to induce a paradigm shift may emerge if RCTs validate incremental HD safety. Ultimately, this could spur a holistic reconfiguration of HD norms, influenced by robust scientific evidence, clinical practice, stakeholder engagement and policy reform (Fig. 3).

**Table 2:** RCTs comparing incremental HD with conventional HD.

Study	Investigator(s)	Target total sample	Residual kidney function eligibility	Primary outcome	Country	Trial registry
IHDIP [16]	Javier Deira, Miguel A. Suárez-Santisteban *et al.*	152	Kru ≥4 mL/min/1.73 m^2^ (1 HD/week) KRU ≥2.5 to <4 mL/min/1.73 m^2^ (2 HD/week)	Patient survival	Spain	NCT03239808
REAL LIFE [1]	Francesco G. Casino, Carlo Basile, Loreto Gesualdo *et al.*	116	Kru ≥3.0 mL/min/1.73 m^2^ (1 HD/week) Kru ≥1.5 mL/min/1.73 m^2^ (2 HD/week)	Survival of kidney function. ‘Loss of residual kidney function’ defined as UOP <200 mL/24 h	Italy and Poland	NCT04360694
Initiating renal replacement therapy through incremental HD [17]	Fernández M. Lucas *et al*.	88	Kru ≥2.5 mL/min/1.73 m^2^ (2 HD/week)	Loss of residual kidney function, defined as UOP <100 mL/24 h, 12 months after the initiation of HD therapy	Spain	NCT03302546
Impact of incremental versus conventional initiation of HD on residual kidney function [18]	Raja M. K. Kamal, Ken Farrington, Andrew Davenport, Enric Vilar *et al.*	180	Kru ≥3.0 mL/min/1.73 m^2^ (2 HD/week)	Proportion of patients with Kru ≥2 mL/min/1.73 m^2^ at 6 months	UK	NCT03418181
INCH-HD [S22]	Peter Kerr, David Johnson, Andrea Viecelli *et al.*	372	UOP ≥0.5 L/24 h (2 HD/week)	Kidney-specific component of KDQOL-SF36 at 6 months (noninferiority)	Australia, New Zealand, $\& $ Canada	AKTN 20.04
INCHVETS [S23]	Kamyar Kalantar-Zadeh, Csaba P. Kovesdy, Mark L. Unruh *et al*.	252	UOP >0.5 L/day and Kru >3.0 mL/min (2 HD/week)	Physical Component Score of KDQOL-SF36 at 12 months (superiority)	USA	NCT05465044
TwoPlus [S24]	Mariana Murea, Peter Kotanko *et al*.	350	UOP >0.5 L/24 h and Kru >3.5 mL/min/1.73 m^2^ (2 HD/week and adjuvant pharmacotherapy^a^)	Composite: all cause ED visits, hospitalizations or death at 2 years (noninferiority)	USA	NCT05828823

^a^Adjuvant pharmacotherapy encompasses oral loop diuretics, sodium bicarbonate, and potassium binders.

ED, emergency department; KDQOL-SF, Kidney Disease Quality of Life Short Form; UOP, urine output.

Why are RCTs the more potent catalyst for healthcare transformation? Critiques of observational studies primarily target physician-induced selection bias and occult confounding factors, potentially inflating the perceived efficacy of certain treatments. Yet much of dialysis practice is substantially directed by findings of observational studies due to a scarcity of clinical trials in this field. RCTs, where conducted, have considerably influenced practices. In the field of HD, many treatment strategies initially supported by observational data were later refuted by clinical trials. It is worth noting that such trials have catalysed paradigm shifts in HD dosing, haemoglobin targets and timing of HD initiation (Table 1). Thus, learning from historical observational studies scrutinized by RCTs, the nephrology community has reserved judgment on the effects of incremental HD on hospitalization rates, patient survival and renal function variation.

The viewpoint defaming observational studies while exalting RCTs as the ‘gold standard’ is, however, increasingly contested. Strategically conducted observational studies deploying advanced biostatistics afford cost-effectiveness, timely results and diverse patient representation, potentially rivalling their randomized peers. Recent large-scale analyses in non-nephrology spheres found negligible treatment effect differences between well-conducted observational studies and RCTs. Undeniably, contemporary observational studies utilizing methods such as propensity score matching certainly have enhanced optimism regarding the effectiveness of incremental HD [19]. Nonetheless, persistent large discrepancies between observational studies and RCTs have remained noted in studies involving sicker populations, including nephrology studies.

An ethical argument against assigning individuals with sufficient residual kidney function to conventional HD has been raised [20]. Indeed, while some groups of nephrologists have aptly adopted incremental HD, this is by no means a globally embraced practice. In ongoing RCTs, the study enrolees are or will be those already recommended treatment with conventional HD by their healthcare provider because of the many as-of-yet unsurmounted roadblocks to incremental HD.

Limitations of clinical trials are to be acknowledged. Clinical trials are costly, demand significant time and effort, and require navigating systemic and bureaucratic hurdles that act like friction forces. Results from RCTs must not be taken at face value. Details such as data quality, statistical power, participant selection, randomization stratification factors, follow-up duration and analytic strategies must be transparently communicated. Investigative rigor in tracking treatment crossovers, collecting variables associated with both nonadherence and clinical outcomes, and comparison with real-world HD nonadherence, will be crucial to deduce the generalizability of RCT results. Furthermore, results from one RCT often necessitate further validation through additional trials in different patient populations or healthcare settings.

The onus is on the scientific community to employ advanced scientific tools and compile substantial, authentic evidence. This marks not a conclusion, but a pivotal initiation. RCTs should prepare targeted data collection on clinical outcomes, patient-reported outcomes, intervention fidelity, and the administrative cost of incremental HD. Demonstrating clinical parity between incremental and conventional HD could reduce overtreatment and optimize resource use while affording better patient quality of life. Understanding these outcomes across varied scales and practices will yield insights for scalability while fuelling momentum to tackle ensuing challenges.

To achieve broad-spectrum adoption of incremental HD, it will be necessary to redesign interdependent facets of dialysis care. Dynamic educational methods on incremental dialysis will need to reach a range of healthcare providers, such as physicians, advanced practice providers, nurses and dialysis dieticians. Improvements should permeate quality metrics for monitoring and reporting residual kidney function in tandem with its integration into dialysis prescriptions. The need to establish capable IT infrastructures within the realm of dialysis digital health records that calculate and integrate kidney function measurements in dialysis dosing is significant. An optimized dialysis reimbursement policy to accommodate individualized care while maintaining the economic health of the dialysis workforce is a prerequisite to render incremental HD available for all patients who would benefit. Hopefully, data from RCTs on the effectiveness of incremental HD will spur a multidimensional paradigm shift involving all relevant stakeholders. Above all, while undertaking an intensive study of incremental HD, whether it involves prospective cohorts or RCTs, we must prevent history from repeating itself and ensure the principle shaping dialysis care is empirical evidence of individualized care.

## THE MODERATORS’ VIEW

In closing, the moderators note that the realm of healthcare is perpetually evolving, with clinical trials serving as the bedrock upon which medical advancements are validated. These trials should be meticulously designed to test the efficacy and safety of new treatments, interventions and protocols. In the specific context of incremental HD and PD, ongoing trials are being conducted to assess its viability and benefits over conventional, thrice-weekly regimens. Investigators overseeing these trials are tasked with a responsibility that goes beyond mere observation of results. They must ensure that the data derived from these trials is clinically relevant. While preliminary results may suggest benefits, such as reduced mortality or improved cardiovascular outcomes, these findings must be interpreted cautiously. It is indeed essential to consider factors such as the study population's size and diversity, the trial's duration and the specific endpoints being measured. A critical aspect of this cautious approach is the need for further validation across different patient populations and to explore the acceptance by patients, in particular when it comes to increasing the weekly frequency. Incremental dialysis, be it HD or PD, may show promise in a controlled trial setting with a homogeneous patient group, but its effectiveness and safety must be proven in a broader demographic. This includes patients of varying ages, ethnicities and comorbid conditions. Additionally, healthcare settings play a significant role in the applicability of trial results. The infrastructure, resources and staff training in a high-end, urban medical centre may differ drastically from those in rural or under-resourced clinics. In other instances, such as Germany, whole reimbursement systems would need to be altered if incremental HD or PD are proven effective and safe (currently, the weekly flat reimbursement requires 3 sessions per week or a minimum Kt/V otherwise, financial cuts would ensue). Hence, what works in one setting may not transfer directly to another without adjustments and accommodations. Robust evidence is needed to pave the way for the widespread adoption of incremental dialysis. This evidence must convincingly demonstrate that this approach can optimize treatment by aligning it more closely with the patient's needs. It should also show a reduction in overtreatment—avoiding unnecessary dialysis sessions that may not contribute to better outcomes and could even lead to complications or diminished quality of life. Moreover, there is an economic dimension to consider. Healthcare resources are finite, and their judicious use is paramount. Incremental dialysis has the potential to preserve these resources by reducing the frequency of dialysis sessions, thus saving on materials, machine time and staffing. This could lead to significant cost savings for healthcare systems already burdened by the high expenses associated with kidney failure treatment. Improving patients’ quality of life is perhaps the most crucial aspect of any new treatment protocol. Dialysis is a life-sustaining treatment for those with kidney failure, but it comes with a substantial burden, both physically and psychologically. Incremental dialysis could mitigate this by offering a more personalized treatment schedule that allows for greater flexibility and less disruption to patients’ lives.

In conclusion, while the promise of incremental dialysis is significant, the path to its integration into standard care practices still needs rigorous scrutiny and validation. The moderators of this debate maintain that only through thorough examination and cross-contextual validation can we ensure that incremental dialysis provides tangible benefits across the spectrum of patient populations and healthcare environments, ultimately leading to an evidence-based shift in treatment paradigms that enhances patient care and optimizes resource utilization.

## Supplementary Material

sfae020_Supplemental_File
