# Supplementary material for: Incremental dialysis: two complementary views
Source: Clin Kidney J. 2024 Feb 5;17(2):sfae020. doi: 10.1093/ckj/sfae020 (PMC10894032; doi:10.1093/ckj/sfae020)
Supplement: sfae020_Supplemental_File [file sfae020_supplemental_file.docx]

**Supplementary Material**

**Supplementary References in Table 1**

S1. Aoun M. A Cost-Saving Hemodialysis Scheme in Limited-Resource Settings. Kidney Int Rep 2022;7(6):1437-1438. DOI: 10.1016/j.ekir.2022.04.001.

S2. Lin X, Yan Y, Ni Z, et al. Clinical outcome of twice-weekly hemodialysis patients in shanghai. Blood Purif 2012;33(1-3):66-72. DOI: 10.1159/000334634.

S3. Lin X, Gu L, Zhu M, et al. Clinical Outcome of Twice-Weekly Hemodialysis Patients with Long-Term Dialysis Vintage. Kidney Blood Press Res 2018;43(4):1104-1112. DOI: 10.1159/000491566.

S4. Mukherjee T, Devi G, Geetha S, Anchan NJ, Sankarasubbaiyan S. A Comparison of Practice Pattern and Outcome of Twice-weekly and Thrice-weekly Hemodialysis Patients. Indian J Nephrol 2017;27(3):185-189. DOI: 10.4103/0971-4065.202844.

S5. Nieves-Anaya I, Várgas MB, Mayorga H, García OP, Colín-Ramírez E, Atilano-Carsi X. Comparison of nutritional and hydration status in patients undergoing twice and thrice-weekly hemodialysis: a silent drama in developing countries. Int Urol Nephrol 2021;53(3):571-581. DOI: 10.1007/s11255-020-02697-3.

S6. Panaput T, Thinkhamrop B, Domrongkitchaiporn S, et al. Dialysis dose and risk factors for death among ESRD patients treated with twice-weekly hemodialysis: a prospective cohort study. Blood Purif 2014;38(3-4):253-62. DOI: 10.1159/000368885.

S7. Stankuviene A, Ziginskiene E, Kuzminskis V, Bumblytė I. Impact of hemodialysis dose and frequency on survival of patients on chronic hemodialysis in Lithuania during 1998-2005. Medicina (Kaunas, Lithuania) 2010;46:516-21. DOI: 10.3390/medicina46080074.

S8. Parker TF, III, Husni L, Huang W, Lew N, Lowrie EG. Survival of hemodialysis patients in the United States is improved with a greater quantity of dialysis. Am J Kidney Dis 1994;23(5):670-680.

S9. Held PJ, Port FK, Wolfe RA, et al. The dose of hemodialysis and patient mortality. Kidney Int 1996;50(2):550-556.

S10. Port FK, Ashby VB, Dhingra RK, Roys EC, Wolfe RA. Dialysis dose and body mass index are strongly associated with survival in hemodialysis patients. J Am Soc Nephrol 2002;13(4):1061-1066.

S11. Eknoyan G, Beck GJ, Cheung AK, et al. Effect of dialysis dose and membrane flux in maintenance hemodialysis. N Engl J Med 2002;347(25):2010-2019.

S12. Foley RN, Parfrey PS, Harnett JD, Kent GM, Murray DC, Barre PE. The impact of anemia on cardiomyopathy, morbidity, and and mortality in end-stage renal disease. Am J Kidney Dis 1996;28(1):53-61. DOI: 10.1016/s0272-6386(96)90130-4.

S13. Besarab A, Bolton WK, Browne JK, et al. The Effects of Normal as Compared with Low Hematocrit Values in Patients with Cardiac Disease Who Are Receiving Hemodialysis and Epoetin. The New England Journal of Medicine 1998;339(9):584-590.

S14. Seliger SL, Weiss NS, Gillen DL, et al. HMG-CoA reductase inhibitors are associated with reduced mortality in ESRD patients. Kidney Int 2002;61(1):297-304. DOI: 10.1046/j.1523-1755.2002.00109.x.

S15. Wanner C, Krane V, Marz W, et al. Atorvastatin in patients with type 2 diabetes mellitus undergoing hemodialysis. N Engl J Med 2005;353(3):238-248.

S16. Abra G, Kurella Tamura M. Timing of initiation of dialysis: time for a new direction? Curr Opin Nephrol Hypertens 2012;21(3):329-33. DOI: 10.1097/MNH.0b013e328351c244.

S17. Cooper BA, Branley P, Bulfone L, et al. The Initiating Dialysis Early and Late (IDEAL) study: study rationale and design. Perit Dial Int 2004;24(2):176-81.

S18. Locatelli F, Carfagna F, Del Vecchio L, La Milia V. Haemodialysis or haemodiafiltration: that is the question. Nephrol Dial Transplant 2018;33(11):1896-1904. DOI: 10.1093/ndt/gfy035.

S19. Peters SA, Bots ML, Canaud B, et al. Haemodiafiltration and mortality in end-stage kidney disease patients: a pooled individual participant data analysis from four randomized controlled trials. Nephrol Dial Transplant 2016;31(6):978-84. DOI: 10.1093/ndt/gfv349.

S20. Blankestijn PJ, Vernooij RWM, Hockham C, et al. Effect of Hemodiafiltration or Hemodialysis on Mortality in Kidney Failure. N Engl J Med 2023;389(8):700-709. DOI: 10.1056/NEJMoa2304820.

S21. Kimachi M, Onishi A, Tajika A, Kimachi K, Furukawa TA. Systematic differences in effect estimates between observational studies and randomized control trials in meta-analyses in nephrology. Sci Rep 2021;11(1):6088. DOI: 10.1038/s41598-021-85519-5.

S22. INCremental Dialysis to Improve Health Outcomes in People Starting Haemodialysis (INCH-HD). <https://ctv.veeva.com/study/incremental-dialysis-to-improve-health-outcomes-in-people-starting-haemodialysis-inch-hd>.

S23. Incremental Hemodialysis for Veterans in the First Year of Dialysis (IncHVets) (INCHVETS). <https://classic.clinicaltrials.gov/ct2/show/NCT05465044>.

S24. Incremental Hemodialysis: The TwoPlus Trial. <https://classic.clinicaltrials.gov/ct2/show/NCT05828823>.
